# Microbiome and Cognitive Impairment: Can Any Diets Influence Learning Processes in a Positive Way?

**DOI:** 10.3389/fnagi.2019.00170

**Published:** 2019-06-28

**Authors:** Michal Novotný, Blanka Klimova, Martin Valis

**Affiliations:** ^1^Biomedical Research Centrum, University Hospital Hradec Kralove, Hradec Kralove, Czechia; ^2^Department of Management, Faculty of Informatics and Management, University of Hradec Kralove, Hradec Kralove, Czechia; ^3^Department of Neurology, Faculty of Medicine and University Hospital Hradec Kralove, Charles University in Prague, Hradec Kralove, Czechia

**Keywords:** microbiome, diet, cognition, learning, SCFA, antibiotics, neurological disorders

## Abstract

The aim of this review is to summarize the effect of human intestinal microbiome on cognitive impairments and to focus primarily on the impact of diet and eating habits on learning processes. Better understanding of the microbiome could revolutionize the possibilities of therapy for many diseases. The authors performed a literature review of available studies on the research topic describing the influence of human microbiome and diet on cognitive impairment or learning processes found in the world’s acknowledged databases Web of Science, PubMed, Springer, and Scopus. The digestive tube is populated by billions of living microorganisms including viruses, bacteria, protozoa, helminths, and microscopic fungi. In adulthood, under physiological conditions, the intestinal microbiome appears to be relatively steady. However, it is not true that it would not be influenced, both in the positive sense of the word and in the negative one. The basic pillars that maintain a steady microbiome are genetics, lifestyle, diet and eating habits, geography, and age. It is reported that the gastrointestinal tract and the brain communicate with each other through several pathways and one can speak about gut-brain axis. New evidence is published every year about the association of intestinal dysbiosis and neurological/psychiatric diseases. On the other hand, specific diets and eating habits can have a positive effect on a balanced microbiota composition and thus contribute to the enhancement of cognitive functions, which are important for any learning process.

## Introduction

The intestinal microbiome is a diverse community of intestinal microorganisms. For a long time, it seemed unlikely that a microbiome could also be responsible for processes outside the digestive tract. However, it has been shown that the composition of intestinal microbiome affects the entire spectrum of physiological processes or the development of the immune system. The optimal microbiome composition thus contributes to overall health (Al-Asmakh and Hedin, 2015; McKenney and Pamer, 2015). An increasing number of research studies demonstrates the enormous importance of the “gut-brain” axis and suggest that triggers for a variety of neurological diseases can be found in the gastrointestinal tract (Turnbaugh et al., 2009; Fasano, 2012; Hsiao et al., 2013; Bauer et al., 2016; Schroeder and Bäckhed, 2016; Dinan and Cryan, 2017).

The microbiome consists of milliard living microorganisms and as such, it has 100 times more genes than the human genome. The microbiome evolves with its human host in a symbiotic relationship. The development of the microbiome as a finely tuned ecosystem depends on a number of factors. The most important are: type of childbirth delivery, eating habits (diet, lifestyle; Daniel et al., 2014; David et al., 2014; Bruce-Keller et al., 2015; Magnusson et al., 2015), it is influenced by infection, stress, genetic predisposition, or person’s age (Yatsunenko et al., 2012; David et al., 2014).

The aim of this review is to summarize the effect of human intestinal microbiome on cognitive impairments and to focus primarily on the impact of diet and eating habits on learning processes. Better understanding of the microbiome could revolutionize the possibilities of therapy for many diseases.

## Methods

The authors performed a literature review of available human and in one part also animal studies on the research topic describing the influence of human microbiome and diet on cognitive impairment or learning processes. The research studies were selected on the basis of research topics such as microbiome, neurological disorders, cognitive impairment, dementia, diet, learning processes found in the world’s acknowledged databases Web of Science, PubMed, Springer, and Scopus. The end of the search period is limited by March 2019. Altogether 531 were identified in all these databases. After removing duplicates and titles/abstracts unrelated to the research topic, 178 English-written studies remained. Of these, only 51 articles were relevant to the research topic. These research studies were classified according to their relevancy. The information found in the selected studies on microbiome and cognitive impairment was carefully evaluated and it is described and discussed in the following sections.

## Microbiome

The digestive tube is populated by billions of living microorganisms in varying degrees of intensity (Blekhman et al., 2015; Bohórquez and Liddl, 2015; Koppel and Balskus, 2016). Most of them are found in our colon (up to 10^14^), which is considered an extremely complex ecosystem including viruses, bacteria, protozoa, helminths, and microscopic fungi (Bäckhed et al., 2015). In this review article, only the human intestinal bacterial microbiome is discussed (Ley et al., 2006; Qin et al., 2012).

The density of the microbiome in the digestive tract varies by location (at least in the stomach, most in the colon). Based on rRNA sequencing, 52 bacterial strains are currently defined, seven of which colonize the human intestinal tract. Interestingly, of these seven strains, 97% of the microbiome consists of only four strains—Firmicutes, Actinobacteria, Bacteroidetes, Proteobacteria (Tsai et al., 2019).

It is believed that the fetus in the uterus is free of bacteria, and only when it is born, it is inhabited by various strains of bacteria and viruses. However, according to available data, it is highly dependent on the way in which childbirth is conducted. If the delivery is through natural pathways, the main component of the neonatal intestinal microbiome is the bacteria corresponding to the mother’s vaginal microbioma, i.e., the Lactobacillus and Prevotella strains. While if the labor is performed by the so-called cesarean section, the intestine is preferably colonized by the bacteria of the genera Staphylococcus and Corynebacterium. In addition, it was found that children born by cesarean section are at greater risk of developing autoimmune diseases (Bäckhed et al., 2015). Intestinal microbiome is well adapted to external influences during the first 3 years of development and is also prone to undesirable changes that may be caused by diet or antibiotic use (Yatsunenko et al., 2012; David et al., 2014). Research studies state that it is this period of life that is most important to form a healthy intestinal microbiome (Yang et al., 2016). In animal models, antibiotic administration or drastic changes in dietary habits have been shown to lead to a predisposition to a behavioral disorders, depression, and anxiety at a later age.

In adulthood, under physiological conditions, the intestinal microbiome can be said to be relatively steady in terms of both quantity and diversity. However, it is not true that it would not be influenced, both in the positive sense of the word and in the negative one. The basic pillars that maintain a steady microbiome of the individual are: genetics, lifestyle, diet and eating habbits, geography, and age (de La Serre et al., 2010; Yatsunenko et al., 2012; David et al., 2014; Noble et al., 2014, 2017a). Reduced diversity or insufficient microbiome is associated with many diseases (Lozupone et al., 2012; Lloyd-Price et al., 2016; Oh et al., 2016).

## Microbiome-Gut-Brain Axis

The term “gut-brain axis” generally appears in the literature, which includes afferent and efferent neural connections, endocrine, immune and metabolic signals. This means that the gut not only receives regulatory signals from the central nervous system (CNS), but it is also able to send signals to the brain, and the brain receives them. Based on the information published so far, it is very likely that this concept can be extended to the “microbiome-gut-brain” axis, as even the intestinal microbiome can affect the brain (Bercik et al., 2011; Davari et al., 2013; Hsiao et al., 2013; Bruce-Keller et al., 2015). Both the peripheral nervous system modulation and the CNS may be included among the intestine microbial abilities and their metabolites, i.e., influencing the brain development and brain function. This two-way communication system maintains normal organism homeostasis. Changes in microbiota leading to dysregulation of the gastrointestinal system, central/autonomic nervous system, or immune system may be one of the causes of various diseases (Turnbaugh et al., 2009; Fasano, 2012; Hsiao et al., 2013; Bauer et al., 2016; Schroeder and Bäckhed, 2016; Dinan and Cryan, 2017). The brain and intestine are connected by several physiological pathways, including neuronal, endocrine, immune and metabolic pathway.

### Neuronal Pathway

GIT regulation takes place at four levels. At the local level, *via* the enteric nervous system (ENS) containing mainly sensory and motor neurons; at the level of prevertebral ganglia that pass information from the ENS to the CNS (Furness et al., 2014). Another level is the CNS, which, after receiving signals from the periphery and evaluating them, sends instructions to effector cells in the GIT. The last level is specific brain nuclei. The vagus nerve is the direct nerve junction of the CNS and ENS. If bacterial products (endotoxins, inflammatory cytokines, TNF-α) through the ENS stimulate the vagus nerve, affecting the CNS, which is very important for learning and memory-related processes (Mulak and Bonaz, 2004; Sudo et al., 2004; Bravo et al., 2011; Gareau et al., 2011; Forsythe et al., 2014).

### Endocrine Pathway

Enteroendocrine cells are dispersed in the intestinal epithelium. If desired, these cells secrete hormones and other signal peptides. Secretion occurs in response to various luminal stimuli. For example, bacterial by-products have the ability to stimulate enteroendocrine cells. It has been found that if the composition of the intestinal microbiota changes, there will be changes in neuropeptides and neurotransmitters. Possible neuroactive molecules activated in the intestine include, for example, serotonin, GABA, catecholamines, melatonin, acetylcholine, histamine, dopamine (Barrett et al., 2012). These molecules can regulate inflammation, affect stress and anxiety reactions, emotions and mood, play a role in learning and creating memory tracks (O’Mahony et al., 2015). A brief review of the function of serotonin, according to the location of its receptors in the CNS/ENS, and in view of its affectivity and cognition (Table 1).

**Table 1:** Serotonin receptor subtypes through gut-brain axis (adjusted according to O’Mahony et al., 2015).

Subtype	CNS function	ENS function
5-HT1A	mood, cognition, pain, sleep, neuroendocrine function	release of mediators, degranulation of enteric mast cells
5-HT2A	sleep, hallucinations	contraction of gut smooth muscle
5-HT2B	hyperphagia, grooming behavior	contraction of gut smooth muscle, visceral sensitivity
5-HT2C	mood, food intake	N/A
5-HT3	emesis, pain, release of other neurotransmitters	pain, secretory and motor responses
5-HT4	mood, cognition	contraction of smooth colonic muscle, prokinetic effect, neurotransmitter release
5-HT5A	mood, sensory perception, neuroendocrine functions	N/A
5-HT6	mood	N/A
5-HT7	mood, sleep	muscle relaxation

### Immune Pathway

An important role of intestinal mucosa is to mediate adaptive immunity because it gets into primary contact with a large number of specific antigens. These specific antigens are PAMPs (pathogen-associated molecular pattern molecules). These are lipids, lipopolysaccharides and lipoproteins, which are part of the bacterial wall. PAMPs are recognized by pattern recognition receptors (PRRs). Activated PRRs provide increased cytokine and interferon production through antigen presenting cells. PRRs include, for example, transmembrane TLRs (Toll-like receptors). TLRs are localized in cells that are part of innate immunity (macrophages, epithelial cells, adipocytes). However, we also find them in cells of acquired immunity (B-lymphocytes, T-lymphocytes or dendritic cells). TLRs-mediated signaling results in the induction of dendritic cells and subsequent cytokine production (Thomas et al., 2017; Caputi and Giron, 2018).

### Metabolic Pathway

Short-chain fatty acids (SCFAs), acetate, propionate, and butyrate are among the substances that are produced by the gut microbiome and can affect CNS functions. SCFAs have anti-inflammatory functions that induce due to binding to a specific G protein-coupled receptor (Macfarlane and Macfarlane, 2011). In studies conducted in mice, butyrate was found to regulate energy homeostasis, stimulate leptin production in adipocytes, and cause secretion of several neuropeptides. Butyrate also has anti-inflammatory effects. SCFAs increase the release of serotonin, i.e., they affect behavior and mood, as confirmed by *in vitro* studies.

## Microbiome and Cognitive Impairment

New evidence is published every year about the association of intestinal microbiome, dysbiosis and neurological/psychiatric diseases. The facts relate to both the risk of developing the disease and its progression. Very often, neuro(auto)immune, neurodegenerative or psychiatric diseases are mentioned, such as multiple sclerosis, neuromyelitis optics, Parkinson’s disease (PD), Alzheimer’s disease (AD), amyotrophic lateral sclerosis, anxiety, schizophrenia, autism. Cognitive impairment may occur to a greater or lesser extent with these diseases. While the effect of dieting on cognitive function (see below) has been studied for a long time, the effect of microbiome on cognition is still partially shrouded in mystery (Gareau et al., 2011; Bajaj et al., 2012; Desbonnet et al., 2015; Dinan et al., 2015; Fröhlich et al., 2016; Parashar and Udayabanu, 2016).

Research studies have increasingly put emphasis on the influence of microorganisms on host behavior and its cognitive function. Experiments on germ-free animal models show the appearance of behavioral disorders and reduced cognitive functions. The first animal study has shown that anxiety-like behavior can be achieved by modulating the microbiome. Infection with *Citrobacter rodentium* in combination with acute stress has resulted in memory failure in mice. Interestingly, in control mice, this dysfunction was prevented by the prophylactic administration of probiotics prior to the infection itself (Gareau et al., 2011; Liang et al., 2015). There is evidence that donor animal-like behavior was demonstrated in the target animal when transferring the fecal transplantation from the donor animal to the target animal (Bercik et al., 2011).

For cognitive processes such as memory and learning, the proper functioning of the HPA axis is absolutely necessary. It has been reported that microorganisms (specifically Bifidocaterium) can modulate this axis—under stress, sterile mouse corticosteroid levels and adrenal cortex hormones are much higher than those of conventional microbial mice. Other agents very important for learning and memory processes are brain derived neurotrophic factor (BDNF), NMDA-receptors (N-methyl-D-aspartate), and c-fos (Wu et al., 2008; Stefanko et al., 2009; Intlekofer et al., 2013). These molecules are reduced in sterile mice. The effect of serotonin on cognition and socialization is well known and therefore the effect of kynurenine pathway metabolites is not so surprising. A well-studied example of the effect of dysbiosis on cognitive function is liver encephalopathy (that can lead to dementia), which is somewhat positively affected by oral antibiotic therapy (Bajaj et al., 2012; Ahluwalia et al., 2016). On the contrary, there exist studies that have demonstrated that the administration of antibiotics in mice causes cognitive impairments. Experiments were performed in mice immediately after weaning and in adult animals. The reduction in the number of microorganisms and their reduced diversity due to antibiotic therapy has always been associated with a decrease in hippocampal BDNF expression. However, it is not known whether the effects of antibiotics on cognitive function are only transient. It is clear from the above how the intestinal microbiome is a fragile community of microorganisms (Fröhlich et al., 2016).

A recently cross-sectional study conducted in Japan showed a comparison of the gut microbiome between demented and non-demented patients and demonstrated two major clusters of microbial taxa. The results were surprising: the number of Bacteroides (enterotype I) was lower and the number of “other” bacteria (enterotype III) was higher in demented than non-demented patients. Multivariable analyses revealed that lower prevalence of Bacteroides and a higher prevalence of “other” bacteria were associated with higher odds ratios than the traditional dementia biomarkers ApoE ε4, SLI and high VSRAD score (Saji et al., 2019).

Interesting facts are brought by research studies on the influence of the use of dairy probiotic cultures among healthy volunteers. They have seen a change in activity in the brain regions responsible for cognitive function, these areas being under serotonergic innervation. Thus, the administration of serotonin and tryptophan precursor for modulating learning processes might be significant (Tillisch et al., 2013).

In the context of inappropriate eating habits, excess energy intake and lifestyle without enough physical activity, one can talk about the obesity epidemic in the western world. The findings of the research studies have shown that obesity and intestinal dysbiosis go hand in hand. In addition, in these studies, it was suggested that the intestinal microbiotic composition also manifested itself in results of cognitive and other tests. Persons with a higher incidence of actinobacteria (non-obese) were found to have better results at the speed of movement, better attention, and better cognition scores. Mechanistic pathway is not yet studied, however, there is a link between diet, microbiome, inflammation and resulting cognitive dysfunction in obese patients (Pistell et al., 2010; Puig et al., 2012; Herculano et al., 2013; Tillisch et al., 2013; Camer et al., 2015).

## Diets and Cognitive Dysfunction

Research studies show that diet and eating habits have both long-term and short-term effects on people’s cognitive functions (Mahoney et al., 2005; Prado and Dewey, 2012). Diet and proper eating habits influence person’s cognition already in prenatal age since diet plays a key role in the maturation of vital organs and the establishment of neuronal connections (Moody et al., 2017). Research indicates that diseases (e.g., AD or autism) connected with cognitive impairments and learning disabilities have their etiology in an early life (Moody et al., 2017). Thus, maternal diet can have a long-term effect on child’s cognitive development. Particularly two extremes are inappropriate; malnutrition or food deficit and excessive intake of saturated fat and refined carbohydrates (Mahoney et al., 2005; Prado and Dewey, 2012).

Malnutrition is typical of the so-called developing countries such as India, where researchers discovered among 8-year-old children suffering from chronic malnutrition that 19% of them find it difficult to read simple sentences like “I like dogs” or “The sun is hot,” 12.5% make a mistake when they are asked to write a simple sentence and 7% make mistakes while responding to simple mathematics such as eight minus three (Angre, 2019). In addition, Wang C. et al. (2016) in their study among 1,366 Chinese adults born between 1950 and 1964 found out that malnutrition is associated with overall and specific cognitive decline, affecting selective attention and response inhibition particularly. Nevertheless, food deficiency is also connected with socio-economic status even in affluent countries where children from deprived backgrounds receive food of low quality, with fewer micronutrients (e.g., iron or iodine), fewer calories, but with higher fat (Darmon and Drewnowski, 2008). As Ross (2019) state food consumption is vital to the brain to make the right amount of amino acids and choline—important precursor molecules to make the brain function normally. Furthermore, researchers emphasize the importance of habitual breakfast for school children since such breakfast has a positive effect on pupils’ academic performance, mainly on task-performance behavior in the classroom (Adolphus et al., 2013).

On the contrary, the excessive intake of saturated fat and refined carbohydrates is typical of western diet (Reichelt et al., 2017), which also has a negative impact on cognitive functioning since high fat and sugar change intestine bacteria colonies and increase intestinal permeability and lower blood brain barrier. This develops a vulnerability to the influx of toxins from circulation to the brain, which results in cognitive dysfunction (Noble et al., 2017b).

Research reveals that diets, especially therapeutic for some pathological conditions such as irritable bowel syndrome or neurological disorders, are also effective in enhancing cognitive functions (Lichtwark et al., 2014; Reddel et al., 2019). This concerns mainly specific dietary regimens such as low-fermentable, oligo-, di-, monosaccharides and polyols (low-FODMAPs) and gluten-free (GFD). They seem to be useful for recovery and maintenance of a eubiotic gut microbiota, particularly if supported with probiotics (Reddel et al., 2019).

## Discussion and Conclusion

Neurological diseases are a global problem and the professional public expects them to grow. The rapid lifestyle, the enormous consumption of antibiotics and other eradicating microbial processes that have so far coexisted with humans for millennia, can be summarized as westernization. Westernization is a huge challenge for medical research. The changing intestinal microbiome, its association with neurological diseases, and the association with prolonged life expectancy are beginning to play a significant role for society.

A number of animal model studies have been reported to indicate that changes in the intestinal microbiota can be linked to gut-brain axis effect and influencing nutrient absorption, energy distribution or immunity. The opposite situation is with experiments on humans. There is an insufficient number of adequately designed controlled trials leading to a difficult assessment if the microbiome composition can influence cognition and learning processes. Considering the results from animal studies, one can reasonably believe that microbiome could affect gut-brain axis and influence cognition in humans. This means that positive qualitative and quantitative changes in the human gut microbiota might interfere with the onset and development of cognitive impairment and improve learning processes.

More and more studies are focused on TLR receptors and their association with neurological disorders and the microbiome-gut-brain axis. Increasing evidence attributes positive effects to probiotic restoration of disrupted intestinal barriers, stimulation of the human mucosal immune system, and prevention of growth of pathogenic microorganisms. TLR ligands derived from probiotics appear to have a positive effect on inflammatory production of anti-inflammatory cytokines (Thomas et al., 2017; Caputi and Giron, 2018).

On the basis of published studies, the use of probiotics can be particularly recommended to support cognitive functions. Most work has been done on animals, but the portability of results to humans seems realistic. *B. longum*, *B. breve*, *B. infantis*, *L. helveticus*, *L. rhamnosus*, *L. plantarum*, and *L. casei* were most effective at improving CNS function (anxiety, depressive, affective, stress, memory; Tsai et al., 2019; Wang H. et al., 2016).

Tryptophan resp. the serotonin system plays an irreplaceable role in the gut-brain axis. Given the diversity of serotonin functions throughout the human body, it does not seem realistic yet to treat individual diseases caused by the serotonin deregulation. It is both a heterogeneity of the disease and a low level of exploration of the area. Because preclinical data strongly favor this neuromediator, the connection of microbiome-gut-serotonin-CNS to researchers seems to be a major challenge, but studies are now in the process of adding that systems such as GABA are completely unexplored and open to cognition (O’Mahony et al., 2015).

Moreover, specific diets (low-FODMAPs and GFD) and eating habits can have a positive effect on a balanced microbiota composition and thus contribute to the enhancement of cognitive functions, important for any learning process.

Probably the biggest controversy can bring contradictions in the results of individual studies. Some articles describe sensational discoveries, others exploring virtually the same, finding changes barely statistically detectable. It is not uncommon to find contradictory results. The reasons may be different. Cognition disorders and learning difficulties are often multi-etiological and the same cognitive impairment in one patient may be caused by other causes, whether genetic, metabolic, or immunological, to the other individual. Another fact is that cognition disorders can be masked, for example, by training to improve test results; cognition and learning can also disrupt mood disorder already altered by possible psychotherapy or life circumstances. False negative results can be caused by an inappropriate selection of control objects (for example, when examining microbiomes of family members as controls). Other patient groups (e.g., due to possible microbiological contamination in the hospital environment) may also be an inappropriate control. The choice of the subjects themselves should also be carried out wisely, especially in the case of small numbers. Some of the microbiome sequencing studies operate with a small number of subjects, and in addition, from the same geographical area. Therefore, it cannot be ruled out that in other areas, countries, cultures with other dietary and hygiene habits, dysbiosis at the microbiome level may be manifested differently. For a complete list of problems in understanding the analysis, it is worth noting that stool samples, which most studies explore, do not have to show the real representation of species in the gut. For example, there are mucinophilic bacteria, mutualists, and possibly pathogens, who are only minimally involved in excretion and thus invasive methods are needed to investigate them.

In conclusion, the authors are convinced that by changing the lifestyle, including changes in dietary habits, can positively affect the cognitive brain functions and learning-related processes, especially by supporting the physiological mechanisms discussed in this mini-review. In addition, recent research (Toman et al., 2018) shows that other interventions such as physical activities or cognitive training are also an integral part of cognitive processes.

## Author Contributions

MN, BK, and MV equally contributed to the drafting, analyses and final version of the whole manuscript. All authors read and approved the final manuscript.

## Conflict of Interest Statement

The authors declare that the research was conducted in the absence of any commercial or financial relationships that could be construed as a potential conflict of interest.
